# Hard X-ray Fourier transform holography at free electron lasers source

**DOI:** 10.1038/s41598-024-67972-0

**Published:** 2024-07-30

**Authors:** Wojciech Roseker, Rustam Rysov, Wonhyuk Jo, Taito Osaka, André Philippi-Kobs, Leonard Müller, Matthias Riepp, Michael Walther, Alexey Zozulya, Lars Bocklage, Felix Lehmkühler, Fabian Westermeier, Daniel Weschke, Michael Sprung, Ichiro Inoue, Makina Yabashi, Gerhard Grübel

**Affiliations:** 1 Deutsches Elektronen-Synchrotron DESY, Notkestr. 85, 22607 Hamburg, Germany; 2 RIKEN SPring-8 Center, Sayo, Hyogo 679-5148 Japan; 3 The Hamburg Centre for Ultrafast Imaging, Luruper Chaussee 149, 22761 Hamburg, Germany; 4 Present Address: European X-ray Free Electron Laser Facility, Holzkoppel 4, 22869 Schenefeld, Germany; 5 Present Address: Sorbonne Université, CNRS, Laboratoire de Chimie Physique - Matière et Rayonnement, LCPMR, 75005 Paris, France

**Keywords:** X-rays, Techniques and instrumentation

## Abstract

We report on the feasibility of Fourier transform holography in the hard X-ray regime using a Free Electron Laser source. Our study shows successful single and multi-pulse holographic reconstructions of the nanostructures. We observe beam-induced heating of the sample exposed to the intense X-ray pulses leading to reduced visibility of the holographic reconstructions. Furthermore, we extended our study exploring the feasibility of recording holographic reconstructions with hard X-ray split-and-delay optics. Our study paves the way towards studying dynamics at sub-nanosecond timescales and atomic lengthscales.

## Introduction

Hard X-ray free-electron lasers are natural sources for investigations of ultra-fast phenomena^1–5^. In particular, ultra-short XFEL pulses, routinely below 100 fs, and the possibility of accessing short wavelengths below 1 Å pave the way towards studying ultra-fast dynamics at atomic lengthscales. Coherence is another very prominent feature of XFELs^6,7^ making them a promising tool to study structures and dynamics of macromolecules and viruses in real space via lenseless coherent X-ray imaging techniques^8^. Several prominent examples of coherent diffractive imaging (CDI) on nanoscale materials^9,10^ and Fourier transform holography (FTH) from magnetic domains^11,12^ and light-induced phase transitions^13^ have been reported. Both techniques strongly rely on the high degree of transverse coherence and combined with ultra-short pulses can enable collecting (quasi-) static snapshots of the sample dynamics within few femtoseconds. While in CDI experiments the phase is reconstructed via an iterative algorithm^14^, the FTH approach, using well-defined reference objects, provides an image of the sample structure by a simple Fourier transform. In recent years, FTH developed into a robust technique with soft X-rays. First demonstrations of femtosecond holographic X-ray imaging has been carried out with soft X-ray FEL pulses^15,16^ followed by other studies on magnetic^11^ and biological systems^17^ . A combined FTH and CDI study performed at a synchrotron radiation source demonstrated the feasibility of hard X-ray imaging with a spatial resolution of 25 nm^18^. Recently, structures of three-dimensional test objects of sub-$\upmu \hbox {m}$ dimensions could be resolved with a resolution better than 3 nm^19^ via CDI.

In FTH imaging the sample and reference objects, e.g. dots, apertures, are illuminated by a coherent beam and the interference pattern i.e., a hologram is formed in the far field. A Fourier transform of the measured hologram gives the autocorrelation term shown in the center of the hologram and the cross correlation terms of the object and the reference dots in the rest of the image. The cross-correlation terms show the object shape and its complex conjugate for each reference dot. The resolution of the object image depends on the size of the reference dots. The smaller the size of the dots the higher is the spatial resolution of the reconstruction at the cost of fringe visibility of the hologram. In contrary to the iterative algorithm method, FTH can be better suited for low-photon statistics^20^

Studying dynamics in a real space via FTH imaging at ultra fast time scales requires very bright and high repetition rate X-ray sources. Pulse repetition rates at XFEL sources, e.g., of 60 Hz at SACLA^2^, 120 Hz at LCLS^1^ and 4.5 MHz at European XFEL^4^ allow reaching up to sub-microsecond time scales in a movie mode, i.e., 16.6  ms, 8.3 ms and 222 ns using the intrinsic source time structure. Accessing shorter time scales can be achieved employing special accelerator techniques^21^ or via crystal-based split-and-delay optics (SDO)^22–27^. Split-and-delay devices, that split a single FEL pulses into two pulses with tunable delay-times, have been successfully demonstrated to operate with soft^28^ and hard X-rays^29–31^. Delay times between the two split pulses from 0 fs up to few nanoseconds have been achieved with fs resolution. However, combining FTH with the split-and-delay technique at FEL sources (split-pulse FTH) has been only successfully demonstrated with soft X-rays so far^28^. Hard X-ray SDO devices consist of wavefront division beam splitter and multiple Bragg crystal reflectors. The beam splitter divides the coherent beam geometrically and can introduce fringes into the beam profile. Coherence properties of the beam passing the split-and delay optics have been investigated using speckle contrast^32^ and hard X-ray interferometry^24^. However FTH imaging together with hard X-ray SDO has not been demonstrated so far.

The challenge for hard X-ray FTH imaging is reduced scattering cross-section compared to soft X-ray FTH. Typically samples and reference structures are also reduced. This results in much smaller scattered intensity at the detector and in consequence reduced signal to noise ratio. On the other, hand employing hard X-rays brings a possibility of achieving higher spatial resolution and investigating thick samples or even buried structures that are not accessible with soft X-rays.

Here, we present a hard X-ray imaging study based purely on FTH at the free electron laser SACLA. We investigated the feasibility of obtaining a single and multi-pulse holograms. By varying the number of FEL pulses on the sample and selecting various attenuations we estimated the adequate fluence for the successful FTH reconstruction using solid state nanostructures. We extended our study by investigating the feasibility of FTH reconstructions from the individual branches of the SDO. We were able to record holographic reconstructions with a resolution of $\approx$ 60 nm.

## Results

**Figure 1:**
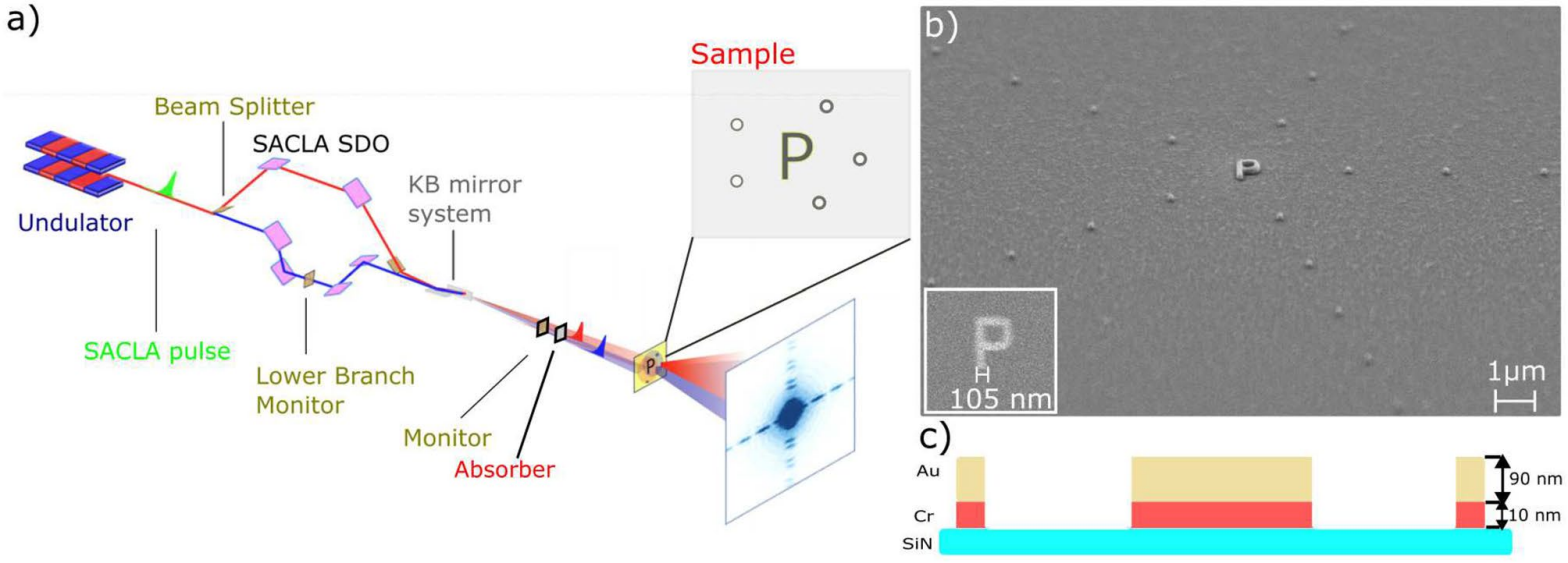
**(a)** Schematics of the hard X-ray FTH experiments using the SACLA SDO system. Each SASE pulse is split into two pulses using the SDO. The pulses are focused with the KB optics and travel in the sample direction with a delay time. Two beams illuminate the sample with a partial overlap. Holograms are collected by the detector. Time delay between the pulse pairs is defined by the SDO system. **(b)** SEM image of the selected sample (object and reference structures i.e., dots) on the silicon nitride membrane. The inset shows the zoom into the sample object (i.e., letter P). **(c)** Detailed cross-section of each sample structure.

**Figure 2:**
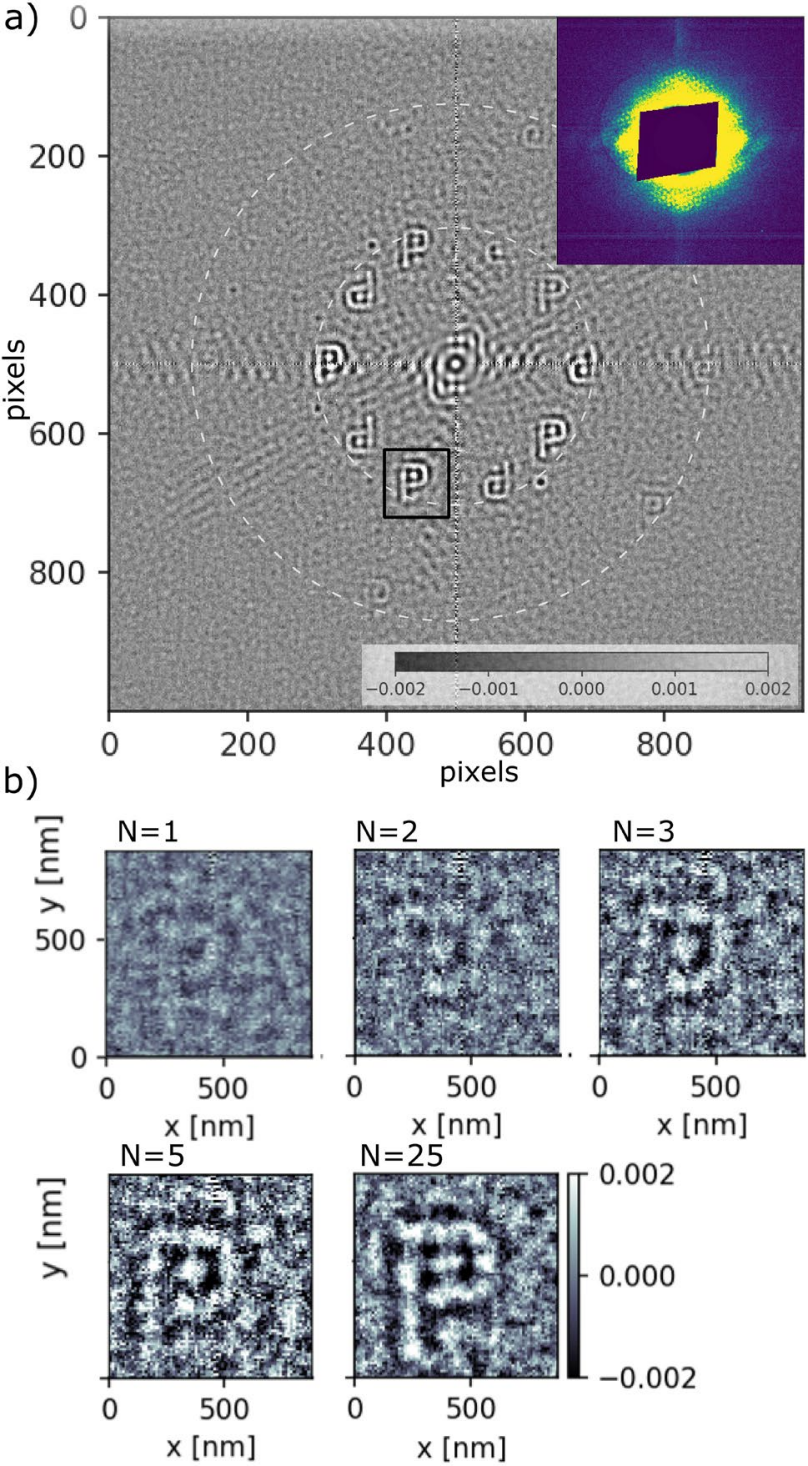
(**a**) Reconstructed sample (real part of the image) obtained from the hologram shown in the inset of the figure. Dark blue central part corresponds to the pixels hidden by the beamstop. Dashed white circles show the location of the reconstructed objects. Black rectangle denotes the cross-correlation location used for the single and multi-pulse analysis. (**b**) Single ($N=1$) and multi-pulse reconstructions as a function of the number of pulses *N*.

In total 3 P-letter objects denoted as P$_{1}$, P$_{2}$, P$_{3}$ have been independently investigated at SACLA using SDO system, as shown in Fig. 2a. The experimental details are given in the Methods section. Figure 1b shows the SEM image of selected $P_{1}$ structure together with the reference dots placed on concentric circles with the radii of multiples of r$_{P}={2}\,{\upmu \hbox {m}}$. The average width of each P structure was 105 nm. As the FEL beam is fully transverse coherent, the number of reference dots used in the measurements was defined by the size of the beam. Figure 2a shows the reconstructed real part of the sample object P$_1$. The central part of the hologram is shown in the inset of the figure. The interference pattern in the obtained hologram arises from the arrangement of the reference dots and the sample object. The recorded image is a result of a mesh scan around the sample object covering the first and second reference dot-ring. This procedure allowed us to find available reference structures and find an optimal beam illumination position for the sample measurements. In total 493 single pulse images have been collected with an average pulse energy of ${0.12}\,{\upmu \hbox {J}}$ passing the lower branch of the split-and-delay system. As the size of the objects is very small the scattered intensity for the strongest pulses is $\approx$ 6 $\times$ 10$^3$ photons at the detector position. Despite of averaging reconstructions from all measured positions the letter *P* is clearly recognisable at locations closest to the center of the image. Reconstructions of the cross-correlations at a larger distance (${4}\,{\upmu \hbox {m}}$) from the center (i.e., the second ring of reference structures) show a very low visibility due to lower statistics. Figure 2b shows an individual reconstruction of letter-P$_{1}$ as a function of the number of pulses *N* illuminating the sample. The visibility of the reconstructed sample varies with number of pulses *N*. We quantified the visibility by calculating variance in the region of interest (ROI1) covering P-letter and compared to the background variance (ROI2) (see Fig. 3b) in Supplementary Information). For low pulse numbers (N <6) the reconstruction the sample shape is recognisable and the visibility values stays above the noise. Here, the single pulse FTH was obtained with a pulse energy of $~{0.16}\,{\upmu \hbox {J}}$. Thanks to a higher statistics with $N>24$ the reconstruction rises clearly above the noise.

**Figure 3:**
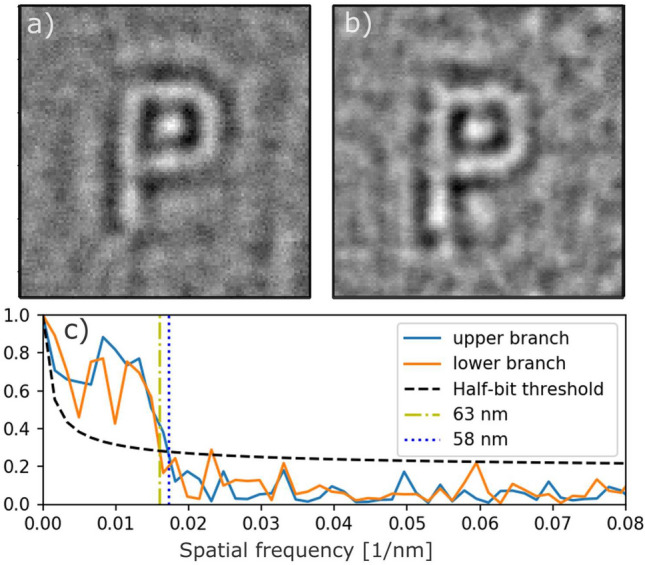
Reconstruction (real part of the image) obtained with the (**a**) upper and (**b**) lower branch of SDO system, respectively. (**c**) FRC analysis of the reconstructions shown in (**a**) and (**b**). Black dashed line corresponds to half-bit threshold^33^ Vertical blue and yellow lines denote the resolution of the reconstructions of 58 nm and 63 nm for the upper and lower branch, respectively.

The quality of multi-pulse reconstructions has been investigated separately when either the upper or lower branch of the split-and-delay was blocked. The holograms were collected at a single sample position and correspond to incident average energy of 0.07${\upmu \hbox {J}}$/pulse and ${0.05}\,{\upmu \hbox {J}}$/pulse for the lower and upper branch, respectively. Figure 3 shows the reconstructed images from the P-letter (P$_{2}$). A round feature inside the reconstruction of the letter P is a result of missing data covered by the beamstop (see Supplementary Information). Additionally cross-correlation between higher order reference dots affects the quality of the reconstruction. The resolution of the holographic reconstruction is defined by the speckle size $\Delta _s$, the reference structures size $\Delta _\text {r}$ and the maximum *q* values reached in the experiment according to $\Delta$q = $\pi$/$q_{\text {max}}$ = 11 nm. The speckle size at the detector was larger than the pixel size ($\Delta _\text {px}={50}{\upmu \hbox {m}}$ ). It was obtained via $\Delta _s$ = $\lambda L / b_{\text {s}} = {141}\,{\upmu \hbox {m}}$, where $\lambda$ is the X-ray wavelength. We quantified the resolution of the reconstructions using Fourier ring correlation (FRC) method^33,34^ by correlating two FTH reconstructions resulting from two sets of independent measurements. Each set consisted of data from 40 FEL pulses. The FRC analysis (see Fig. 3) indicates a resolution of 58 nm and 63 nm for the upper and lower branch, respectively. These values are in agreement with the results obtained in the independent studies using synchrotron radiation^35^. Reducing the size of the reference dots should further improve the resolution of the reconstructions^36^. The visibility of the reconstructions can be improved by increasing the pulse intensity unless sample degradation effects occur due to beam induced heating effects. In order to avoid beam induced damage the above measurements were recorded with attenuated beam conditions limiting the maximum pulse energy to ${0.2}\,{\upmu \hbox {J}}$ on the sample (see Supplementary Information). SEM images taken after the FEL measurements (see Fig. 2a,b in Supplementary Information), indicate that the structure of samples *P* $_1$ and *P* $_2$ have not been affected by the FEL beam.

**Figure 4:**
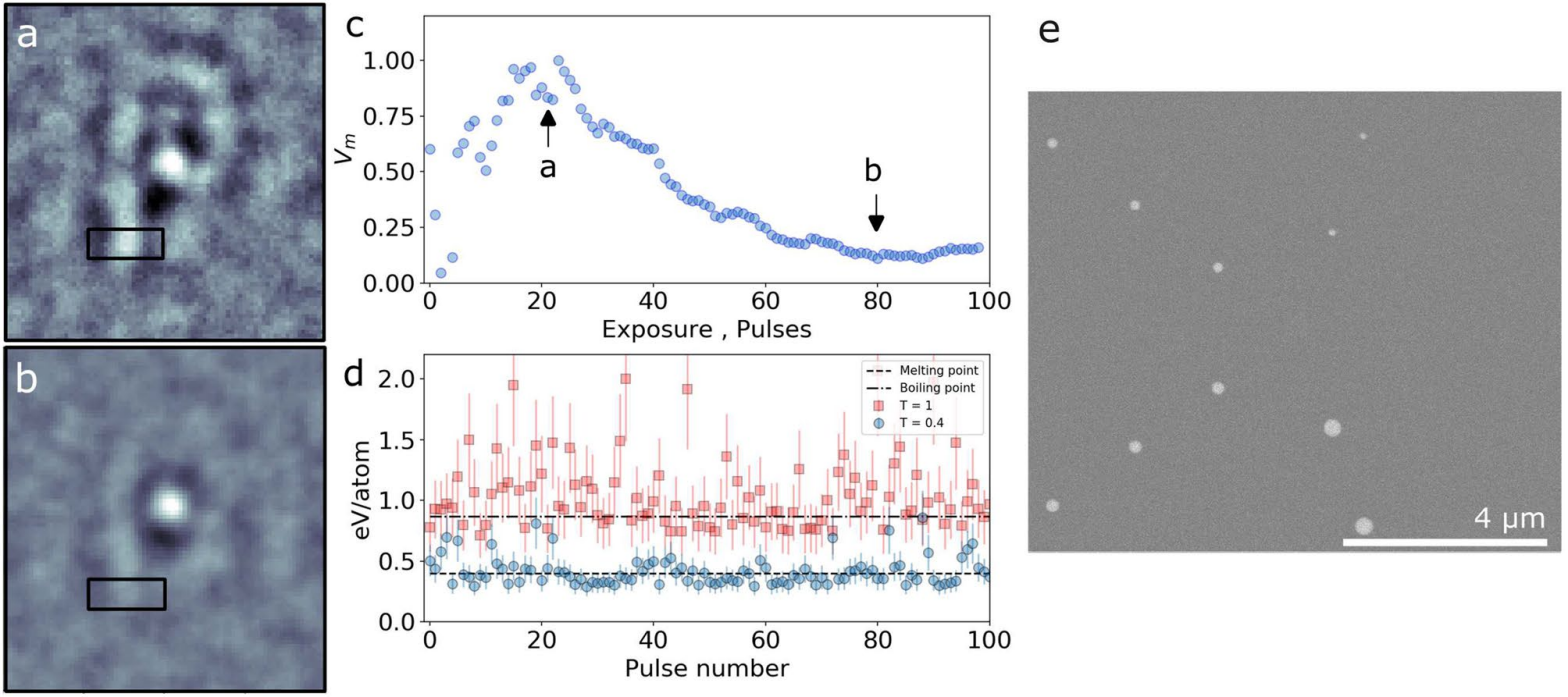
Reconstruction (real part of the image) obtained with a transmission T = 0.4 and exposure of (**a**) 20 FEL pulses and (**b**) 80 FEL pulses. (**c**) Normalized variance obtained from the ROIs as a function of total accumulated pulse energy. The ROIs are denoted by the black rectangles in (**a**) and (**b**). The variance of (**a**) and (**b**) is depicted with the black arrows. (**d**) Calculated maximum energy per Au atom in the sample as a function of pulse number. (**e**) SEM image of the sample taken after the FEL study.

Extremely bright nature of FEL pulses can cause significant heat deposition at the sample position, causing non-equilibrium electronic heating followed by thermalization with the lattice and heat diffusion to the surrounding (e.g., substrate). Here, we have investigated the maximum fluence on sample P$_3$ by varying transmission values of the attenuator and total detector integration time (i.e, number of pulses). Pulse energies measured at the sample varied between 0.15 and ${1}\,{\upmu \hbox {J}}$ (see Supplementary Information). Figure 4a shows the reconstruction of the sample obtained with attenuator transmission *T* = 0.4 and an exposure of 20 FEL pulses and the mean intensity of ${0.2}\,{\upmu \hbox {J}}$/pulse. Exposing the sample to *N* = 80 pulses (see Fig. 4b) reduces further the visibility of the image. The strong round feature visible in the reconstruction is a result of the cross-correlation between the 1st and 2nd circle of reference dots. Figure 4c shows the visibility V$_{\text {m}}$ of the reconstruction as a function of number of pulses calculated from the variance of the signal within the ROI depicted by the black rectangle in Fig. 4a,b. Here, the visibility is related to the sample structure changes and reduces with sample degradation. The V$_{\text {m}}$ peaks at the value $\approx$ 20 pulses and decreases to 0.1 for exposures above 80 pulses. Thanks to the higher statistics the visibility first increases, however it starts to decrease for larger exposures. This might indicate the beam-induced heating of the sample.

Melting of the sample due to the heat load is expected when the viscous flow time of the sample is shorter as compared to thermal diffusion^37^. The heat dissipation times calculated for Au, Cr and Si in the sample, using finite element analysis (see the Supplementary Information), indicate that the temperature drops back to the room temperature within 100 ns. The values are much shorter than the time separation between subsequent incident FEL pulses ($\approx$ 16.6 ms) and therefore only a single pulse heating (the single branch operation) is taken into account. Figure 4c shows the pulse energy deposited per Au atom in the sample as a function of pulse number (see Supplementary Information). The melting point (0.4 eV/atom^38^) was reached for individual FEL pulses. It can cause the sample to melt and re-crystalize leading to deformations. Measurements without attenuation result in up to ${1.1}\,{\upmu \hbox {J}}$/pulse on the sample, which is sufficient to vaporize the sample (0.87 eV/atom is the boiling point of Au^38^). Figure 4e shows SEM image of the sample $P_{3}$ taken after the FEL studies. The object P evaporated from the SiN surface together with the right row of reference dots.

## Discussion

We have investigated the quality of FTH reconstructions obtained with single and multi pulses from SACLA. Through the use of hard X-rays, we were able to resolve the shape of the sample with the smallest features of $\approx$ 100 nm without any pre-defined assumptions and phase retrieval iterative algorithms. The obtained resolution values of $\approx$ 60 nm obtained with upper and lower branches of the SDO were sufficient to resolve the shape of the object at fluences below ${0.2}\,{\upmu \hbox {J}}$. Sufficiently high visibility of the reconstructions was achieved with 25 FEL SACLA pulses.

Further development of the hard X-ray FTH requires addressing enhancements in the resolution and signal-to-noise ratio. Employing hard X-rays for FTH imaging could provide higher spatial resolution compared to soft X-rays. Here, the resolution of the reconstructions was limited by the size of the reference dots and the beamstop. Utilizing Focused Ion Beam (FIB) as the structuring method promises reduction of the reference dot sizes^39^ to about 10 nm. Improving the signal to noise ratio of the reconstructions without increasing the radiation dose could be achieved by increasing the illumination beam size. As the FEL beam is fully transversely coherent, illuminating a larger number of reference dots is feasible^20^. Other approaches to improve the signal to noise and resolution might include uniformly redundant arrays ^40^, non-iterative Coherent Diffractive Imaging ^41^ or customizable references ^42^. Furthermore, the visibility of the reconstructions can be further improved by holographic aided iterative phase retrieval ^18,43,44^. In the experiment very low wave vector transfer values up to $0.017\, \text {nm}^{-1}$ were covered by the beamstop. In this part, the hologram contains the information on the extended areas in the reconstructed sample. Figure 6 (in Supplementary Information) shows how this effect introduces artifacts in the reconstruction. Use of smaller or semitransparent beamstops or filling the center information by a measurement without the beamstop will further improve the resolution.

Our results have been obtained with individual branches of the split-and-delay optics, which is an important step towards investigating dynamical phenomena via split-pulse FTH^28^. In this technique the two pulses are delayed with respect to each other at the sample. A slight angular mismatch between the two pulses allows to set the beams such that only a particular set of reference dots are illuminated by each pulse separately keeping the overlap on the sample object. The two holograms generated by the pulses are collected within the same exposure of the detector and are completely independent and allow recovering the temporal and spatial information. Integration of the split-pulse method at FELs together with FTH imaging brings several advantages. The two pulses originate from the same electron bunch providing intrinsic synchronization. The time resolution of the measurement depends on the pulse length and the accuracy of time-delay and is independent of X-ray source repetition rate or detector frame rate. In this way structural changes on femto-to picoseconds timescales can be tracked in a real space as a function of the time delay between the pulses, set by the SDO.

The single pulses passing the SDO system were intense enough to induce heating effect in the sample. Reconstructions obtained with pulse energies above ${0.2}\,{\upmu \hbox {J}}$ show structural changes and poor visibility of the sample, indicating ultra-fast structural changes. The split-and-delay techniques provides jitter-free access to X-ray pump X-ray probe experiments within relevant time window from sub-100 fs to few picoseconds. Upon very intense FEL radiation the sample starts to lose large number of band electrons which leads to a weaker scattering and can cause structural changes of the sample^45,46^ that might be investigated by FTH.

Our study is a step towards future use of XFELs and split-and-delay optics to track ultra-fast dynamics. Using hard X-rays together with FTH gives potential of reaching high spatial resolution with smaller reference structures and femtosecond time scales to track, e.g. the evolution of radiation damage on ultra-short time scales, non-thermal phase transitions, thus stimulating new developments in theory and establishing new bridges between experiment and theory.

## Methods

### Experimental

The FEL studies have been carried out at the experimental hutch EH4c at BL3 of SACLA XFEL^2^. Figure 1a shows the sketch of the experimental setup. SACLA operated in SASE mode delivering ultra-short (<10 fs) pulses with pulse energies between 100 and ${330}\,{\upmu \hbox {J}}$ and repetition rate of 30 Hz. The FEL pulses were generated with 9 keV photon energy and monochromatized to the bandwidth of $\Delta \lambda / \lambda = 6 \times 10^{-5}$ with Si(220) crystal optics. The beam was split vertically into two parts using a wave division beam-splitter and delayed with the SACLA-SDO system^24^. The two parts propagate along two unequal paths inside the device defined by perfect Bragg crystals. One part of the beam is guided by the Bragg crystals located in the upper side of the setup (called upper branch). The path of the second part is defined by the Bragg optics of the lower side of the SDO (called lower branch). Both beams were carefully positioned at the sample using a high resolution imager^47^. Low fluence mesh scans of the SiN membrames with the sample objects were utilized to identify the illumination positions on the sample and the reference dots. Kirkpatrick–Baez (KB) mirrors were used to focus the beam to $4 \times {4}\, {\upmu \hbox {m}}^2$ at the sample position. A set of Aluminum attenuators were placed in the beam downstream the SDO to reduce beam heating effects on the sample. Holograms were recorded by MPCCD Octal detector with the pixel size $\Delta _{\text {px}}={50}\,{\upmu \hbox {m}}$ located at 4.1 m distance from the sample position. The detector was operating with 30Hz frame rate. A beamstop was placed in front of the MPCCD detector to block the direct beam.

### Sample

The sample P-letter objects were fabricated by sputter deposition, e-beam lithography and ion beam etching. Figure 1c shows the cross-section of the sample structure. A bilayer of 10 nm-Cr/90 nm-Au was deposited onto 200 nm thick silicon nitride membranes. A negative resist stop was structured by e-beam lithography. All excess metal was removed by Ar ion-beam etching. The residual resist was removed by an oxygen plasma, leaving only the metallic structures on the silicon nitride membrane. In order to speed up the alignment of the sample to the FEL beam, a $50\times {50}\, {\upmu \hbox {m}}^2$ Au square frame was deposited around each sample object, as shown in Fig. 1 (see Supplementary Information).

SEM images of all samples are shown in the Supplementary Information. The average width of the objects is 105 ± 5 nm, as shown in the inset of Fig. 1b. Each sample object is surrounded by positive reference structures (called reference dots). The dots are placed on multiple circles around the sample and generate reference waves for the FTH reconstruction. SEM image of the sample structure, i.e. letter-P with the dots is shown in Fig. 1b. The smallest dots that were fabricated are approximately 80 nm in diameter. The total sample thickness is much smaller than the longitudinal coherence length ($\approx {1.14}\,{\upmu \hbox {m}}$). All samples were pre-characterized at the coherence application beamline P10, PETRA III^35^. Prior to FTH reconstruction, the data have been processed to reduce detector noise and artefacts by subtracting dark images.

### Data

The pixels behind the beamstop were masked. To reduce artifacts appearing in the reconstructions caused by the missing data region of the beamstop area we applied a filter defined by $1- 1/ \sqrt{1+(r/r_\text {B})^{2n}}$, where *r* is a radial coordinate $r_{\text {B}}$ corresponds to a radial size of the beamstop. The factor *n* allows controlling the slope of the filter function close to pixels corresponding to the beamstop edges. The parameters *n* and $r_B$ were set to 7 and 30 pixels, respectively (see Fig. 7 in Supplementary Information). Additionally all the holograms were centered. The visibility of the reconstruction was obtained by calculating the variance of the ROIs shown in Fig. 3 in Supplementary Information.

### Supplementary Information

Supplementary Information.

## Data Availability

Data supporting the findings of this study are available from the corresponding author on reasonable request.
