# Supplementary material for: Hard X-ray Fourier transform holography at free electron lasers source
Source: Sci Rep. 2024 Jul 30;14:17480. doi: 10.1038/s41598-024-67972-0 (PMC11289130; doi:10.1038/s41598-024-67972-0)
Supplement: Supplementary file 1 — Supplementary Information. [file 41598_2024_67972_MOESM1_ESM.pdf]

Hard X-ray Fourier transform holography at Free Electron Lasers source-Supplementary Information

Wojciech Roseker[1,*], Rustam Rysov[1], Wonhyuk Jo[1,5], Taito Osaka[2], André Philippi-Kobs[1], Leonard Müller[1], Matthias Riepp[1,6], Michael Walther[1], Alexey Zozulya[4], Lars Bocklage[1,3], Felix Lehmkühler[1,3], Fabian Westermeier[1], Daniel Weschke[1], Michael Sprung[1], Ichiro Inoue[2], Makina Yabashi[2], and Gerhard Grübel[1,3,5]

[1]Deutsches Elektronen-Synchrotron DESY, Notkestr. 85, 22607 Hamburg, Germany, [2]RIKEN SPring-8 Center, Sayo, Hyogo 679-5148, Japan, [3]The Hamburg Centre for Ultrafast Imaging, Luruper Chaussee 149, 22761 Hamburg, Germany, [4]European X-ray Free Electron Laser, Holzkoppel 4, 22869 Schenefeld, Germany, [5]Present address: European X-Ray Free-Electron Laser Facility, Holzkoppel 4, 22869 Schenefeld, Germany, [6]Present address: Sorbonne Université, CNRS, Laboratoire de Chimie Physique – Matière et Rayonnement, LCPMR, 75005 Paris, France

[*]wojciech.roseker@desy.de

Sample alignment

Figure 1 shows the SiN membrane with the samples. Each sample is located inside of a $50{\times}50\,\mu m^2$ Au frame, that can be easily pre-aligned using an optical microscope. The sample objects were aligned to the X-ray beam with a mesh scan at low fluence pulse conditions. The membrane was translated such that the FEL beam was illuminating the center of the frame, where the sample was positioned.

Samples

Sample objects P_1 P_2 and P_3 were investigated in detail. Figure 2 shows the SEM images of the selected structures measured after the FEL experiment.

Visibilty

The visibility of the reconstructions was quantified by calculating the variance of the selected ROI (see Fig. 2b in the manuscript). Fig 3 shows the variance of the reconstructions obtained from N pulses.

Heat estimation at the sample

We estimated the heating at the sample structure (i.e., Au, Cr and SiN) caused by the intense XFEL pulses. The cross section of the sample object is shown in Fig. 1c (in the manuscript). The absorbed energy per atom was calculated according to [1]

$$E \approx \frac{N E_{ph} \sigma M}{N_a b_s^2},$$

where N_a is Avogadro's number, M is the molar mass, and N is the number of photons in a single FEL pulse and b_s is a beam size. The σ corresponds to the photoabsorption cross-section. For Au $\sigma = 151.7\mathrm{cm}^2/g$ and M = 196.9 g/mol [2].

Fig. 4 shows pulse energies measured at the sample position. We simulated the expected heat dissipation time from the illuminated area by solving the transient heat transfer equation applied for the sample structure shown in Fig. 1c. A finite element transient thermal analysis is conducted using the ANSYS software [3] by approximating the sample structure with finite elements, solving on element nodes and piece-wise interpolating of the field quantity (i.e., temperature). Boundary conditions are applied including a volumetric heat generation to simulate the FEL beam and the interface between the sample and the surrounding medium. The heat values are obtained using a single beam energy of 0.16 µJ and applied within the first 100 fs on the illuminated area taking into account absorption of various elements in the sample. Fig. 5 shows the resulting temperature profile of Au, Cr and SiN in the sample as a function of time.

Simulated FTH reconstructions

Figure 6 a) shows the hologram of the sample object P and reference structures shown Fig. 2. The image comprises of 1000×1000 pixels with q_max= 0.278 nm^{-1}. Resulting holographic reconstruction is shown in Fig. 6c). The effect of the beamtstop masking lower q values q_min = 0.017 nm^{-1} is shown in Fig. 6b). Corresponding reconstruction in Fig. 6d) shows the non-uniform visibility across the P-letter and a round feature in the middle of the reconstruction.

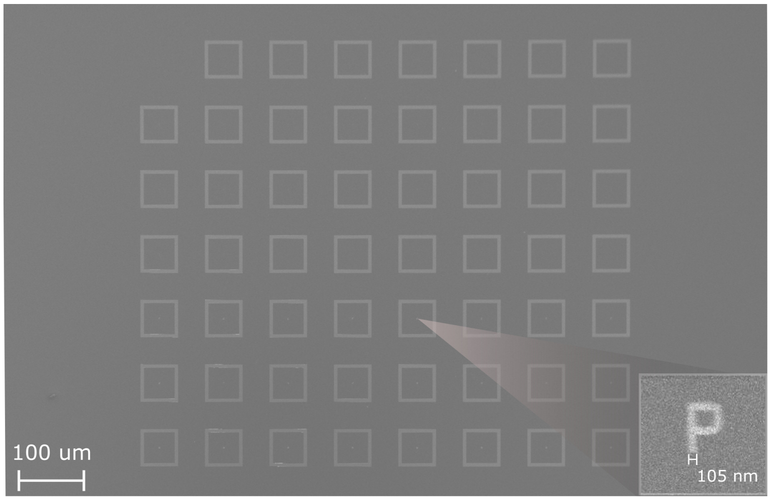

Figure 1: SiN membrane with holographic samples located inside quadratic gold structures. The size of Au structures is $50 \times 50\,\mu m^2$. A typical P-letter structure is shown in the inset.

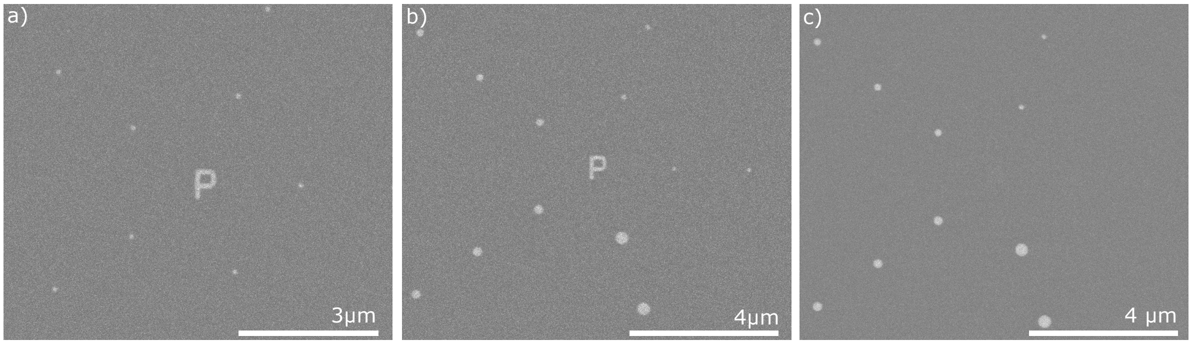

Figure 2: SEM images of the P structures a) P_1 b) P_2 c) P_3 measured after the FTH experiment.

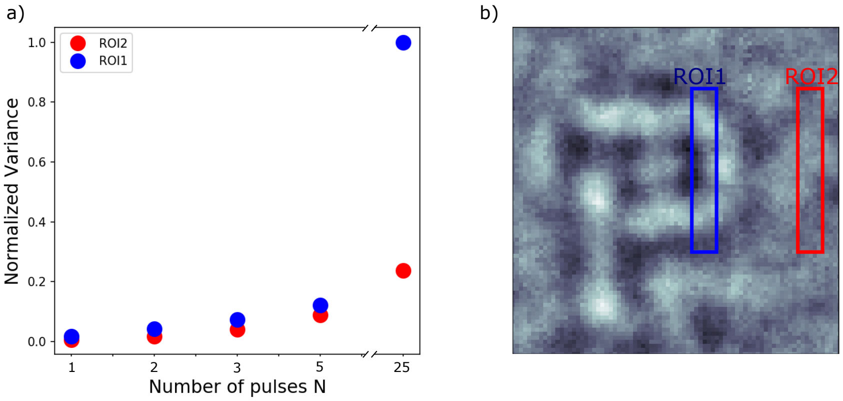

Figure 3: a) Calculated variance of the reconstructed P-letter object (ROI1) and background (ROI2) as a function of the number of accumulated pulses N. b) The position of the ROI1 and ROI2 are denoted by blue and red rectangles, respectively. The size of each ROI is 100×430 nm^2.

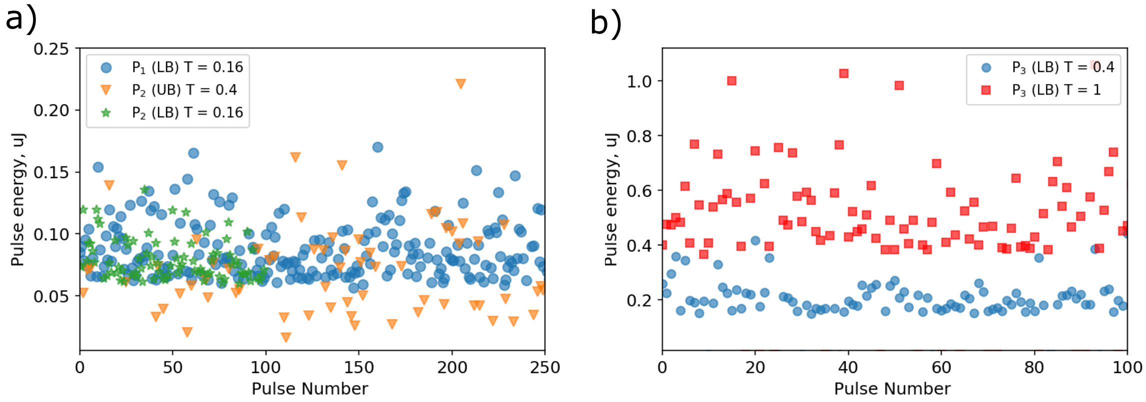

Figure 4: a) Pulse energies at the sample position measured for P_1 and P_2 structures with lower branch (LB) and upper branch (UB) for two attenuator settings. b) Pulse energies measured at the P_3 structure.

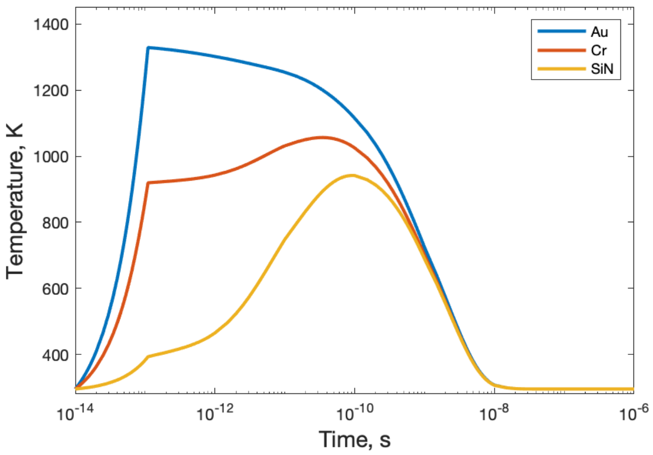

Figure 5: Simulated temperature of Au, Cr and SiN in the sample as a function of time. The pulse energy used in the simulations was 0.16 µJ/pulse

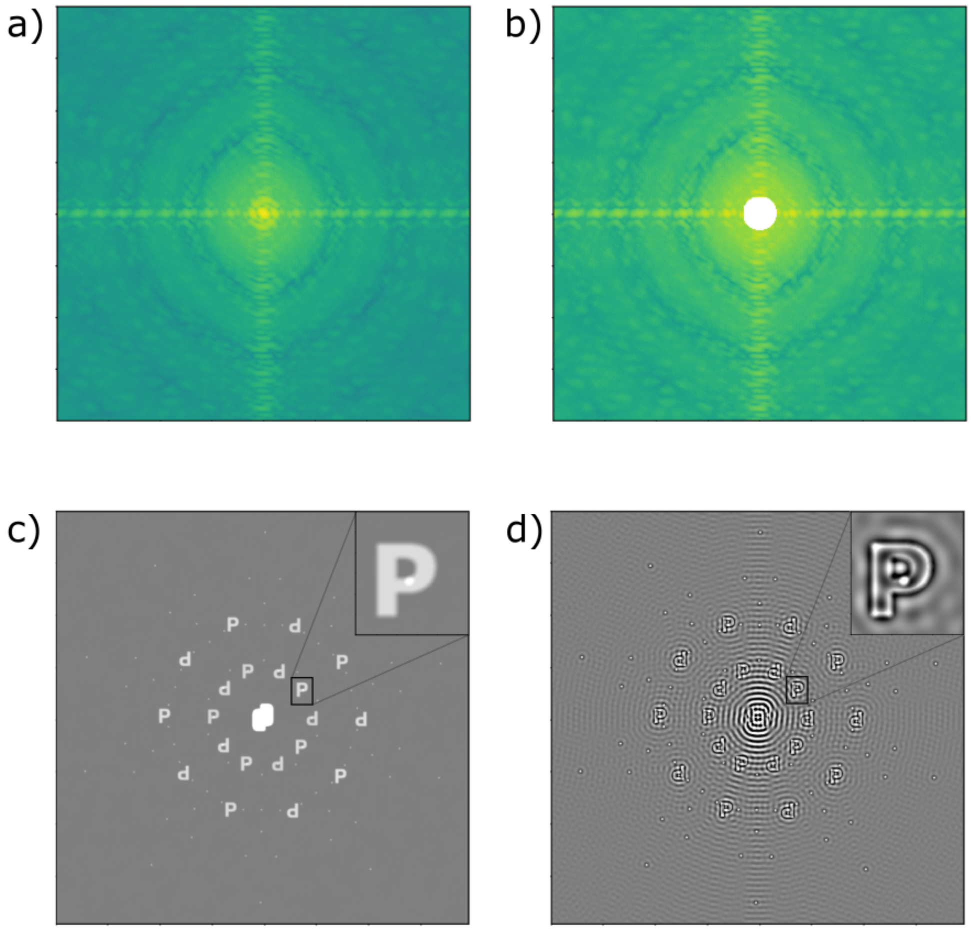

Figure 6: Calculated Fourier transform hologram of the object based on the SEM images shown in Fig. 2 without the beamstop a) and with the beamstop b). To emphasise the effect of the beamstop on the reconstruction, the reference structures are set to point-like structures. Reconstructed images of the corresponding holograms c) without the beamstop and d) with the beamstop. The inset in the figures shows a zoom into the reconstructed object.

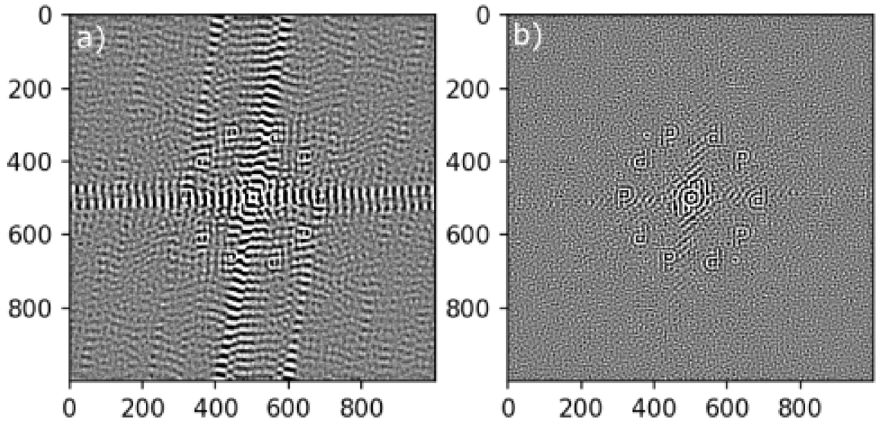

Figure 7: Reconstruction of the sample letter-P exposured to 25 FEL pulses without a) and with the filter b) applied in the analysis.

Bibliography

[1] Jesse N. Clark, Loren Beitra, Gang Xiong, David M. Fritz, Henrik T. Lemke, Diling Zhu, Matthieu Chollet, Garth J. Williams, Marc M. Messerschmidt, Brian Abbey, Ross J. Harder, Alexander M. Korsunsky, Justin S. Wark, David A. Reis, and Ian K. Robinson. Imaging transient melting of a nanocrystal using an X-ray laser. *Proceedings of the National Academy of Sciences*, 112(24):7444–7448, June 2015.

[2] et al. Henke BL. X-ray interactions - photoabsorption, scattering, transmission, and reflection at E=50-30,000 Ev, Z=1-92. *At Data Nucl Data Tables*, 54:181–34, 1993.

[3] F. Yang, D. La Civita, M. Vannoni, and H. Sinn. Damage simulation due to heat load from X-ray FEL beam at European XFEL. *Journal of Physics: Conference Series*, 2380(1):012067, 2022.
